# Enhancing consolidation of a rotational visuomotor adaptation task through acute exercise

**DOI:** 10.1371/journal.pone.0175296

**Published:** 2017-04-13

**Authors:** Blai Ferrer-Uris, Albert Busquets, Virginia Lopez-Alonso, Miguel Fernandez-del-Olmo, Rosa Angulo-Barroso

**Affiliations:** 1 Institut Nacional d’Educació Física de Catalunya, University of Barcelona, Barcelona, Spain; 2 Facultade de Ciencias do Deporte e a Educación Física (INEF Galicia), University of A Coruña, A Coruña, Spain; 3 Kinesiology, California State University, Northridge, Northridge CA, United States of America; German Sport University, GERMANY

## Abstract

We assessed the effect of a single bout of intense exercise on the adaptation and consolidation of a rotational visuomotor task, together with the effect of the order of exercise presentation relative to the learning task. Healthy adult participants (n = 29) were randomly allocated to one of three experimental groups: (1) exercise before task practice, (2) exercise after task practice, and (3) task practice only. After familiarization with the learning task, participants undertook a baseline practice set. Then, four 60° clockwise rotational sets were performed, comprising an adaptation set and three retention sets at 1 h, 24 h, and 7 days after the adaptation set. Depending on the experimental group, exercise was presented before or after the adaptation sets. We found that error reduction during adaptation was similar regardless of when exercise was presented. During retention, significant error reduction was found in the retention set at 1 h for both exercise groups, but this enhancement was not present during subsequent retention sets, with no differences present between exercise groups. We conclude that an acute bout of intense exercise could positively affect retention, although the order in which exercise is presented does not appear to influence its benefits during the early stages of consolidation.

## Introduction

Humans learn and relearn numerous skills throughout their lives. Learning, along with brain function, is known to be influenced by many factors, including engagement in physical activity and a healthy lifestyle [1]. Physical activity, in particular, has been shown to have a positive impact on brain function and cognition [2], with supportive evidence coming from both animal and human studies [1,3–9]. In a recent review, aerobic exercise training programs were shown to improve attention and processing speed, executive function, and memory [10]. However, these benefits seem to depend on characteristics of the exercise, including its mode, intensity, and duration [11]. More specifically, the benefits of exercise are aroused not only by training programs but also by acute bouts of exercise [8]. The evidence suggests that an acute bout of exercise can selectively improve various cognitive processes and enhance memory [11–14].

Despite the growing knowledge base concerning how exercise influences cognitive function, research is scarce regarding how exercise affects specific types of memory. Long-term memory formation requires a two-step process: first, the acquisition (adaptation) of sensory information that will be stored as short-term (working) memory (lasting from seconds to 1–2 minutes) [15]; and second, the consolidation of such memory so it becomes more stable and resistant to perturbation [16,17]. Long-term memory can be split into declarative and non-declarative memory, with the latter being more relevant to learning motor skills [18]. To our knowledge, the following articles have explored the effects of an acute exercise intervention on adaptation and retention of this concrete type of memory. Typically, motor memory consolidation is assessed via retention tests and therefore both terms are used interchangeably in the literature [19].

Statton et al. [20] showed how a moderate-intensity running bout enhanced motor adaptation on the sequential visual isometric pinch task. However, adaptation enhancement did not lead to better retention of the motor skill. Roig et al. [21] studied how an intense acute bout of exercise (cycling) could improve motor adaptation and consolidation of a manual tracking task. Although it did not improve adaptation, exercise had a positive effect on mid-term (24 h) and long-term (7 days) skill retention. Moreover, they found that the presentation order of the exercise in relation to the learning task affected the outcome, with participants who exercised immediately after a learning task showing superior long-term skill retention compared with participants who exercised immediately before. Preceded by Roig’s study, Mang et al. [22] observed that a single bout of intense cycling presented before a sequence-specific motor learning task (continuous tracking) enhanced adaptation and mid-term retention (24 h). Additionally, in a posterior study, the same authors observed that an equal exercise bout improved relearning of a discrete motor sequence task 24 h after adaptation [23]. Overall, Mang’s results suggest that intense exercise could strengthen the adaptation and retention of the motor skill.

The previously cited studies utilized tasks that required some form of motor adaptation while also including [20,22] or not [21] learning of an implicit sequence. Unfortunately, Roig’s and Mang’s studies used similar learning paradigms which limits their generalizability to other procedural learning situations. Moreover, when including the research of Statton et al. (2015), the motor tasks in these studies only required pinch, wrist, or thumb movements in a single direction (e.g. left–right). More complex motor learning paradigms, involving multi-joint and multi-plane movements, are needed to expand our understanding of how exercise can affect the learning process of other gross motor skills. Also, it is unclear how exercise characteristics moderate the exercise effects on adaptation and retention of motor skills. Consequently, a different exercise protocol and learning task is necessary to clarify the extent of exercise-induced benefits on cognitive processes and memory. Lastly, because of the limited evidence regarding the effect of the exercise presentation order in relation to the learning task [21], further research is needed to clarify what presentation order is best to enhance motor learning.

Here, we investigated the effect of running as an acute intense exercise (iE) on the adaptation and retention of a rotational visuomotor adaptation task (rVMA). In addition, we examined the effect of the presentation order of the iE in relation to the rVMA task on retention. We hypothesized that (1) iE would improve the learning rate when presented immediately before the adaptation process of the rVMA task; and (2) iE would improve the rVMA retention process in the short- (1 h), mid- (24 h) or long- (7 days) term. Additionally, we also aimed to explore the effect of the presentation order of the iE and the rVMA task to observe if presentation order may lead to differences on long-term retention.

## Materials and methods

### Participants

In total, 29 adults participated in this study, of whom 21 were males and 8 were females (7 male participants in each group); their mean age, height, and body mass was 21.2 ± 1.9 years, 169 ± 10 cm, and 64.0 ± 80.8 kg, respectively (see Table 1 for participants’ background characteristics). Participants had no prior experience with the proposed learning task (i.e., the rVMA). The exclusion criteria for participation were selected in part to ensure compliance with the exercise protocol and the learning task: left-handedness; low engagement in physical activity; a body mass index above 30 kg/m^2^; below-average intelligence; a self-reported history of neurological, psychiatric, or physical impairment; uncorrected vision worse than 20/20; current medication or recreational drug use that may affect the nervous system or the ability to learn; and smoking. Participants were randomly assigned to one of three groups based on the relationship between the rVMA task and iE: (1) rVMA after exercise (EX–rVMA); (2) rVMA before the exercise (rVMA–EX); and (3) rVMA only (CON). Randomization was checked to ensure in age and fitness level among the three groups, as these factors have been reported to affect how acute exercise alters cognitive performance [24].

**Table 1:** Group characteristics.

	EX_rVMA	rVMA_EX	CON
n	10	10	9
Sex (male/female)	7/3	7/3	7/2
Age (years)	20.9 ± 1.8	20.5 ± 1.8	22.1 ± 1.7
Height (cm)	172.0 ± 12.8	168.7 ± 9.1	168.8 ± 7.9
Body mass (kg)	64.7 ± 11.1	63.8 ± 9.5	63.4 ± 5.6
BMI (kg/m2)	21.7 ± 1.4	22.3 ± 1.8	22.3 ± 2.1
TONI-2-IQ	121.4 ± 6.8	121.3 ± 6.4	125.6 ± 6.4
Estimated VO_2_max (ml/kg/min)	56.9 ± 3.6	55.2 ± 5.5	52.3 ± 8.1
20mSRT HR (bpm)	186.1 ± 9.3	188.1 ± 10.4	186.2 ± 9.7
iE estimated 85% VO_2_max HR (bpm)	182.9 ± 11.4	185.9 ± 12.6	-
iE_estimated 60% VO_2_max HR (bpm)	161.2 ± 14.4	166.4 ± 13.8	-

Abbreviations: BMI = Body mass index; CON = no-exercise group; EX–rVMA = rVMA after exercise group; rVMA = rotational visuomotor adaptation task; rVMA–EX = rVMA before exercise group; TONI-2-IQ = Test of Nonverbal Intelligence version 2– Intellectual quotient; estimated VO_2_max = estimated maximal oxygen uptake; 20mSRT = 20 meter Shuttle Run Test; HR = Heart Rate; iE = intense Exercise. HR during the 20mSRT was calculated as the mean±SD of the last completed minute. HR during the iE was calculated as mean±SD during the last 30 seconds of each estimated 85% or 60% VO_2_max intensity interval.

The study was approved by the Clinical Research Ethical Committee of the Catalan Sport Administration. All participants provided written consent before the study commenced.

### The rVMA task

**Fig 1:**
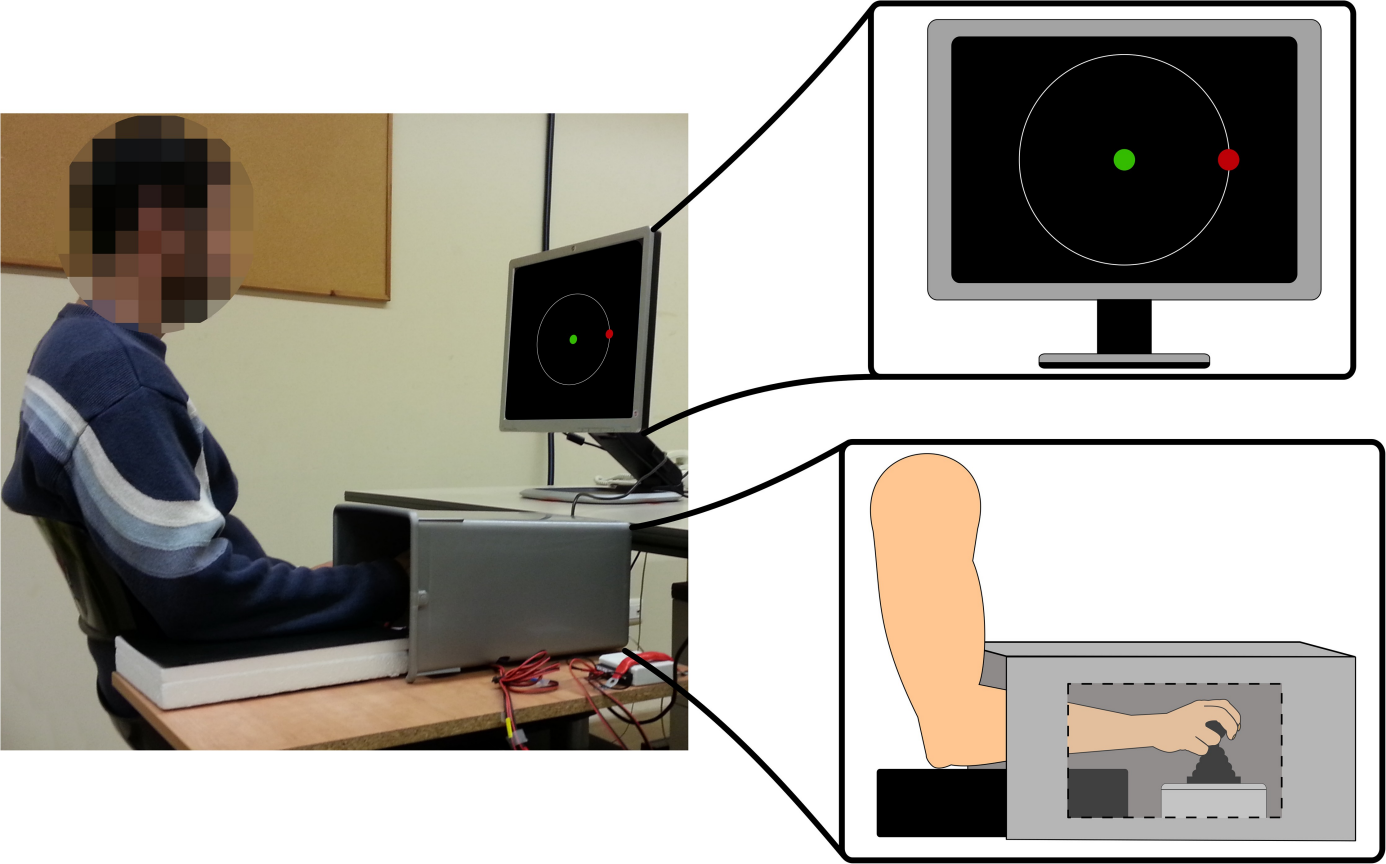
The rotational visuomotor adaptation task (rVMA). Illustration of the setup for the rotational visuomotor adaptation task (rVMA).

The rVMA was conducted in a quiet room. Participants were seated in front of a 19-inch computer screen on which the task was presented. The screen was adjusted to eye level and sited at a distance of 1 meter. Participants’ right arms were then rested over a height-adjustable flat surface to maintain 90° elbow flexion and a comfortable shoulder position. Participants were asked to grasp a non-isometric joystick with their right hand to control a green dot measuring 1 × 1 cm. They were instructed to use a claw-like grip, and to maintain this across all trials (see Fig 1 for a detailed overview of the rVMA setup). An NI USB-6008 card (National Instruments) registered the x, y cartesian coordinates and their corresponding time-points of the joystick movements at a frequency of 120 Hz. Targets randomly appeared every 2 s as red dots (1 × 1 cm) in eight different locations (45°, 90°, 135°, 180°, 225°, 270°, 315°, and 360°, in reference to the vertical midline) at a radius of 13 cm from the center. Each target remained visible for 750 ms. Participants were instructed to start from the center and were encouraged to move the green dot over the target (red dot) and back to the center as fast and as straight as possible in a single movement.

### The iE bout

The acute bout of iE consisted of a 13- min 20-m shuttle run combining a fast and slow speed based on a percentage of the estimated VO_2_max (see *Procedure*): the fast speed corresponded to 85% of the estimated VO_2_max, and the slow speed corresponded to 60% of the estimated VO_2_max. The iE proceeded as follows: 3 min fast + 2 min slow + 3 min fast + 2 min slow + 3 min fast. Exercise protocols of similar intensity have been previously used [21,22] and high intensity interval exercise has recently found to enhance motor learning [25]. Before starting the iE, a 3-min warm-up session was completed (a 2-min slow run and 1-min fast run) to familiarize participants with the iE speeds. A 5-min rest and free stretch period was also permitted before starting the iE. In the case of participants in the EX–rVMA and the rVMA-EX groups, the transition time between the iE and the rVMA was 4 min. We also recorded the participants’ beat-by-beat values for the intervals between electrocardiogram R waves (RR intervals) during the exercise using a Polar RS800CX (Polar Electro) at a frequency of 1 KHz to monitor the exercise intensity. Calculated mean and SD for the heart rate (HR) values of the last 30 seconds of each speed interval are presented in Table 1.

### Procedure

**Fig 2:**
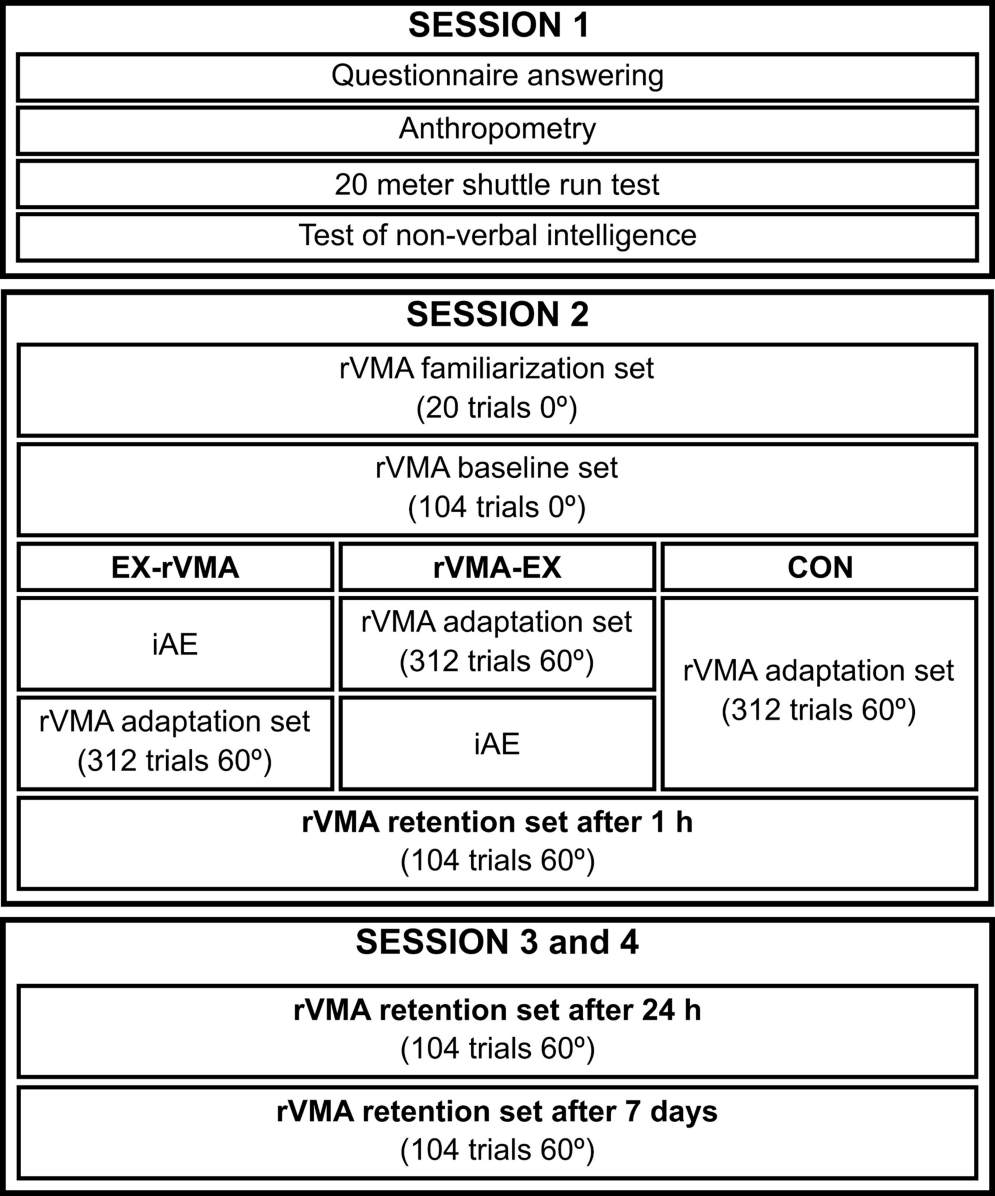
Schematic overview of the experimental procedure.

Four sessions were conducted for each participant (Fig 2).

In session 2, after the rVMA task baseline sets, participants were divided into three groups based on the order of rVMA presentation and whether iE was used. *Abbreviations*: CON = no-exercise group; EX–rVMA = rVMA after exercise group; iE = intense exercise: IDE = initial directional error; RL = Rate of learning; RMSE = root mean squared error; rVMA = rotational visuomotor adaptation task; rVMA–EX = rVMA before exercise group.

In the first session, we reviewed whether the participant met any of the exclusion criteria and assessed their fitness level. Participants were asked to answer a self-report questionnaire related to the exclusion criteria, which included the Physical Activity Readiness Questionnaire (PAR-Q) to assess health status, and the International Physical Activity Questionnaire short version (IPAQ) to assess engagement in physical activity. Basic anthropometry measures (height and body mass) were taken. To assess fitness level (estimated VO_2_max), participants did a 20-m shuttle run test (20mSRT) [26]. During the 20mSRT, beat-by-beat RR values were recorded using a Polar RS800CX (Polar Electro) at a 1 KHz frequency. At the end of the first session, participants undertook the Test of Nonverbal Intelligence version 2 (TONI-2) to assess their intelligence level (TONI-2 Intelligence quotient, TONI-2-IQ). Between the first and the second session, participants were allowed to rest for at least 48 h.

**Fig 3:**
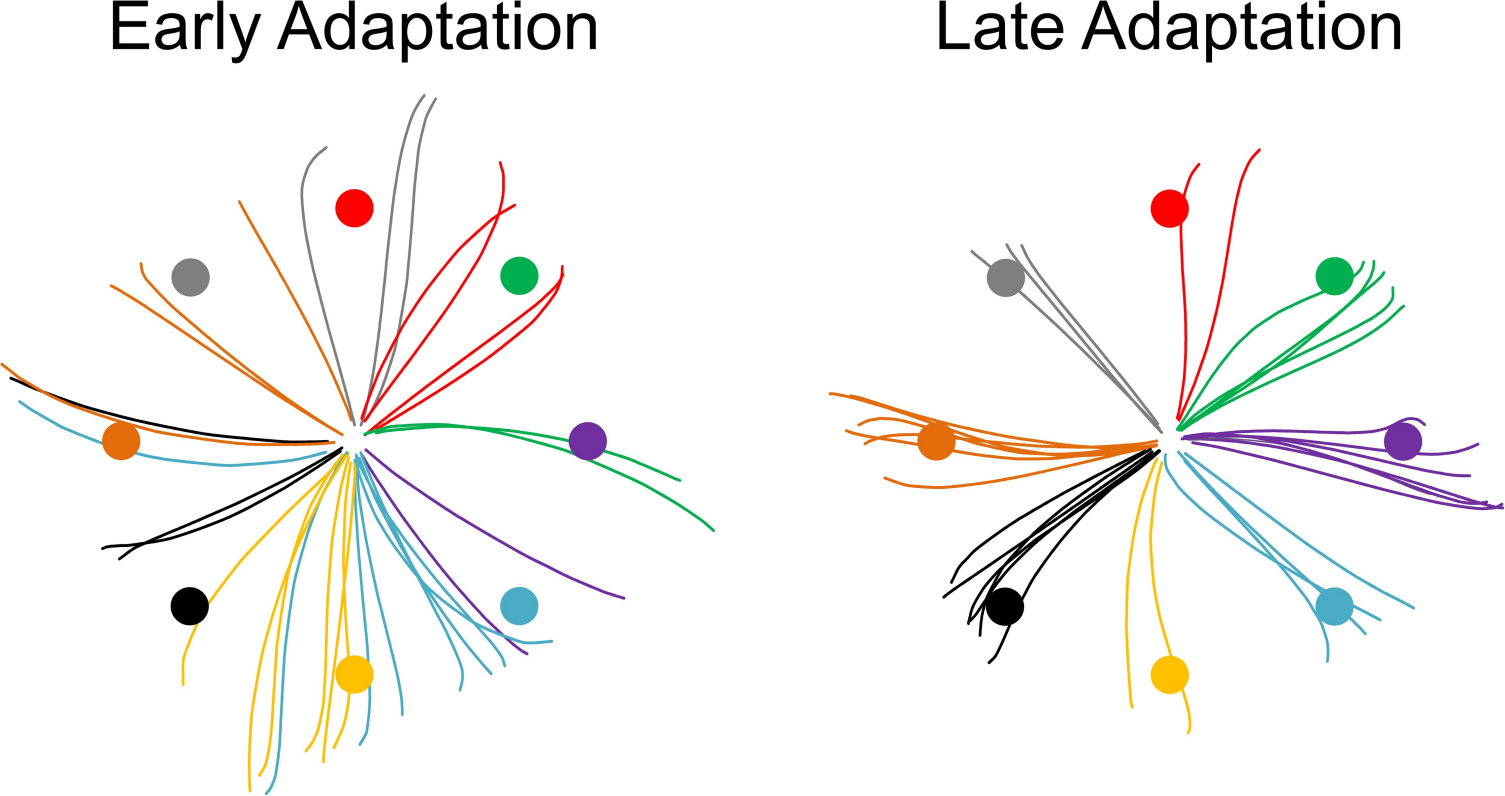
Early and late rVMA cursor trajectories during the adaptation set. The first and last 32 trials of a random participant are shown in the figure. A color has been assigned to each target and its corresponding trajectories to facilitate reading. Greater deviations from the target can be observed during the early adaptation in comparison to the late adaptation. During the test, all targets were presented as red dots.

In the second session, all participants performed the rVMA task, but only the experimental groups performed the exercise protocol. The session started with a familiarization set (20 trials) of non-registered practice in the rVMA task without rotation (0°). When the familiarization set ended, the baseline set was done without rotation (0°; 104 trials). Next, participants did an adaptation set (312 trials) in the rVMA task, with a clockwise rotation of 60° applied to the cardinal coordinates of the cursor movement. Because of this rotation, movements of the hand and joystick appeared on the screen with a clockwise deviation of 60°. An example of the early and late trajectories of the cursor movement during the adaptation set is presented in Fig 3. At this point, the procedure was defined by the participant’s group. The exercise groups did a 13-min iE session before (EX–rVMA group) or after (rVMA–EX group) the adaptation set. Mirroring the rVMA-EX group, the CON group did the adaptation set immediately after the baseline set, but without doing any exercise after completing the adaptation; only reading or holding a conversation was allowed. At 1 hour after the adaptation set, all participants did a 60° clockwise retention set (i.e., the RT1h; 104 trials). During this second session, participants were not allowed to listen to music, do any supplementary exercise, or to sleep.

Sessions three and four were held 24 hours and 7 days after the second session to assess mid- and long-term rVMA retentions, respectively. A 60° clockwise retention set (104 trials) was done in each session (RT24h and RT7d, respectively).

### Data reduction

Custom-made MATLAB R2014b programs (The MathWorks) were used to fit and reduce data. Cartesian positions were low-pass filtered using an eighth-order dual-pass Butterworth filter (cut-off frequency: 12 Hz). Only trials where the start was initially found within 20% of the center-to-target distance were accepted. Movement onset was defined as the nearest point to an outward movement equal to 10% of the center-to-target distance. The movement offset was defined as the point where the speed decreased to 10% of the maximum speed. In addition, we rejected trials in which the traveled distance did not reach 90% of the center-to-target distance. Overall, a total of 110 trials were rejected, which represents a 0.52% of the total executed trials. There were no group differences in the number of trials used for further analyses (Kruskal-Wallis One-Way ANOVA, *p* = 0.478). Finally, all rVMA sets were divided into epochs of eight trials each for analysis purposes.

### Variables

As descriptive variables of movement, we calculated the movement time (MT, ms), the travel distance (TD, cm), and the reaction time (RT, ms), which was defined as the time between target appearance and movement onset. The following movement output error variables were calculated, as presented in previous research [27]: absolute angular initial directional error (IDE, degrees) and root mean squared error (RMSE, cm). IDE was calculated as the difference between the ideal trajectory, defined by the vector between the center point to the target, and the real trajectory, defined by the vector between the center point to the trajectory point at 80 ms after the movement onset. The 80 ms time point was selected to avoid possible corrections guided by visual feedback. RMSE, as straightness measure of the entire movement was calculated, considering the real joystick trajectory and the ideal trajectory (characterized by a straight line), as follows:

$$RMSE=\sqrt{\sum_{i=1}^{N}[{\left(x_{1}-x_{2}\right)}^{2}+{\left(y_{1}-y_{2}\right)}^{2}]\frac{1}{N}}$$

where (x_1_, y_1_) and (x_2_, y_2_) are the coordinates of the real and ideal trajectory, respectively, and N is the number of points in the path.

As seen in other studies [28], we observed an initial rapid change in the error reduction rate followed by a slower decline during the adaptation set. We found that these data were best fitted by a double-exponential decay function of the form:

$$y=a*e^{b*x}-c*e^{d*x}$$

where *y* is the error, *x* is the epoch number and a, *b*, *c*, and *d* are parameters.

To capture the initial rate of learning (RL), we computed the first derivative of the first half of the function and evaluated it at epoch 1, similarly to the method described by Coats et al. [29], for both IDE (RL-IDE) and RMSE (RL-RMSE). All individual correlations were visually inspected for a plateau suggesting that learning was achieved and all correlation values were above 0.8 (RL-IDE *r* = 0.81–0.98; RL-RMSE *r* = 0.87–0.99).

### Data analysis

The assumption of normality was explored with the Shapiro–Wilk test for all variables. As appropriate, variables were transformed or subject to alternative non-parametric tests when the assumption of normality failed. Similarities in age and fitness level (estimated VO_2_max) among groups were explored by one-way analysis of variance (ANOVA). To ensure that the rVMA baseline performance was similar across the three groups, we compared the mean value for each variable (MT, TD, RT, IDE, and RMSE) using ANOVA. The statistical significance was set at *p <* 0.05 for all comparisons.

To address the first hypothesis, Student’s *t-*tests were conducted to analyze the effect of the iE on the average performance of the motor skill adaptation (MT, TD, RT, IDE, and RMSE), comparing those participants who exercised before the rVMA task (i.e. EX–rVMA group [exercise cohort]) to those who did not (i.e. rVMA–EX + CON groups [no-exercise cohort]). When unequal variances were found, Welch’s t test correction was used. We also evaluated the differences between the exercise and the no-exercise cohorts in the rate of learning of the motor skill (i.e., the RL-IDE and RL-RMSE) by using the Mann–Whitney *U* test. In addition, to examine the relation between the degree of learning at 1 h and the adaptation set of both error variables (IDE and RMSE), we used the Pearson correlation coefficient between the end of the adaptation (average of last 4 epochs, 32 trials) and the start of the RT1h (average of first 4 epochs, 32 trials).

Regarding the second hypotheses and the aim to explore the effect of the presentation order of exercise and the learning task, averages were calculated for each variable (MT, TD, RT, IDE, and RMSE) and each rVMA retention set. Differences in the averaged retentions of the motor skill were analyzed by two-way (group × set) repeated-measures ANOVA, with Greenhouse–Geisser sphericity-corrected values reported when appropriate. Where a significant difference occurred, Bonferroni *post hoc* analyses were performed.

Finally, the effect sizes for the different tests were calculated according to Cohen's criteria [30]: *d* was used for *t*-tests (0.2, 0.5, and 0.8 for small, medium and large effects, respectively); *r* for the Mann–Whitney *U* test (0.1, 0.3, and 0.5 for small, medium and large effects, respectively); and η^2^*p* for ANOVAs (0.01, 0.06, and 0.14 for small, medium, and large effects respectively).

## Results

Age and fitness level (assessed by the estimated maximal oxygen uptake [VO_2_max]) along with descriptive and error variables for the rVMA during the baseline set were explored to ensure that there were no baseline differences among groups. Age (F_(2, 26)_ = 2.120; *p* = 0.140; η^2^*p* = 0.140) and estimated VO_2_max (F_(2, 26)_ = 1.433; *p* = 0.257; η^2^*p* = 0.099) parameters revealed no group differences (see Table 1 for means and standard deviation [SD]). Baseline set analysis showed similar rVMA descriptive values among the groups for MT (F_(2, 26)_ = 0.098; *p* = 0.907; η^2^*p* = 0.007), TD (F_(2, 26)_ = 0.320; *p* = 0.729; η^2^*p* = 0.059), and RT (F_(2, 26)_ = 1.677; *p* = 0.207; η^2^*p* = 0.114). In addition, there were no group differences for the error variables, neither for the IDE (F_(2, 26)_ = 00.820; *p* = 0.451; η^2^*p* = 0.059) nor for the RMSE (F_(2, 26)_ = 0.253; *p* = 0.778; η^2^*p* = 0.019) (see Table 2 for means and SD). These results suggested that the randomization procedure was effective in balancing the groups, and that the movement performances were comparable across the three groups in the baseline set of the rVMA task.

**Table 2:** Mean and SD performance values on the rotational visuomotor adaptation task (rVMA) for each group and set.

	EX-rVMA	rVMA-EX	CON
**Baseline**			
MT (ms)	144.49 ± 17.6	143.04 ± 36.0	149.19 ± 37.5
TD (cm)	7.42 ± 0.3	7.49 ± 0.4	7.56 ± 0.4
RT (ms)	346.50 ± 24.2	329.74 ± 17.8	340.37 ± 19.3
IDE (deg)	5.08 ± 1.0	5.79 ± 1.9	5.30 ± 0.5
RMSE (cm)	0.79 ± 0.0	0.81 ± 0.0	0.80 ± 0.1
**Adaptation**			
MT (ms)	154.63 ± 16.6	155.20 ± 37.8	172.01 ± 49.8
TD (cm)	7.43 ± 0.3	7.52 ± 0.4	7.53 ± 0.4
RT (ms)	343.64 ± 37.5	348.97 ± 18.9	350.25 ± 30.3
IDE (deg)	16.07 ± 3.0	14.89 ± 3.0	18.42 ± 3.1
RL-IDE	-11.66 ± 6.1	-12.40 ± 8.3	-11.02 ± 7.9
RMSE (cm)	1.55 ± 0.2	1.45 ± 0.2	1.68 ± 0.3
RL-RMSE	-0.99 ± 0.6	-0.99 ± 0.8	-5.47 ± 13.0
**Retention 1h**			
MT (ms)	148.02 ± 18.2	139.07 ± 25.9	161.71 ± 48.1
TD (cm)	7.37 ± 0.3	7.45 ± 0.4	7.53 ± 0.9
RT (ms)	350.99 ± 33.7	344.03 ± 24.1	346.66 ± 35.4
IDE (deg)	9.70 ± 1.4	9.43 ± 2.2	12.53 ± 2.3
RMSE (cm)	1.08 ± 0.1	1.08 ± 0.2	1.24 ± 0.3
**Retention 24h**			
MT (ms)	148.13 ± 16.3	136.41 ± 28.9	153.58 ± 43.5
TD (cm)	7.60 ± 0.3	7.92 ± 0.4	7.81 ± 0.6
RT (ms)	341.62 ± 29.5	336.62 ± 24.3	335.11 ± 28.3
IDE (deg)	11.66 ± 2.3	11.92 ± 2.2	12.39 ± 2.2
RMSE (cm)	1.27 ± 0.1	1.31 ± 0.2	1.31 ± 0.3
**Retention 7 days**			
MT (ms)	143.46 ± 23.1	138.96 ± 29.8	148.73 ± 41.3
TD (cm)	7.68 ± 0.4	7.85 ± 0.4	7.61 ± 0.4
RT (ms)	338.62 ± 29.2	334.70 ± 23.3	331.26 ± 26.4
IDE (deg)	10.83 ± 2.0	11.56 ± 2.6	11.12 ± 2.3
RMSE (cm)	1.20 ± 0.1	1.28 ± 0.2	1.20 ± 0.2

EX–rVMA = rVMA after exercise group; rVMA–EX = rVMA before exercise group; CON = no-exercise group; MT = movement time; TD = travel distance; RT = reaction time; IDE = initial directional error; RMSE = root mean squared error; RL = rate of learning.

**Fig 4:**
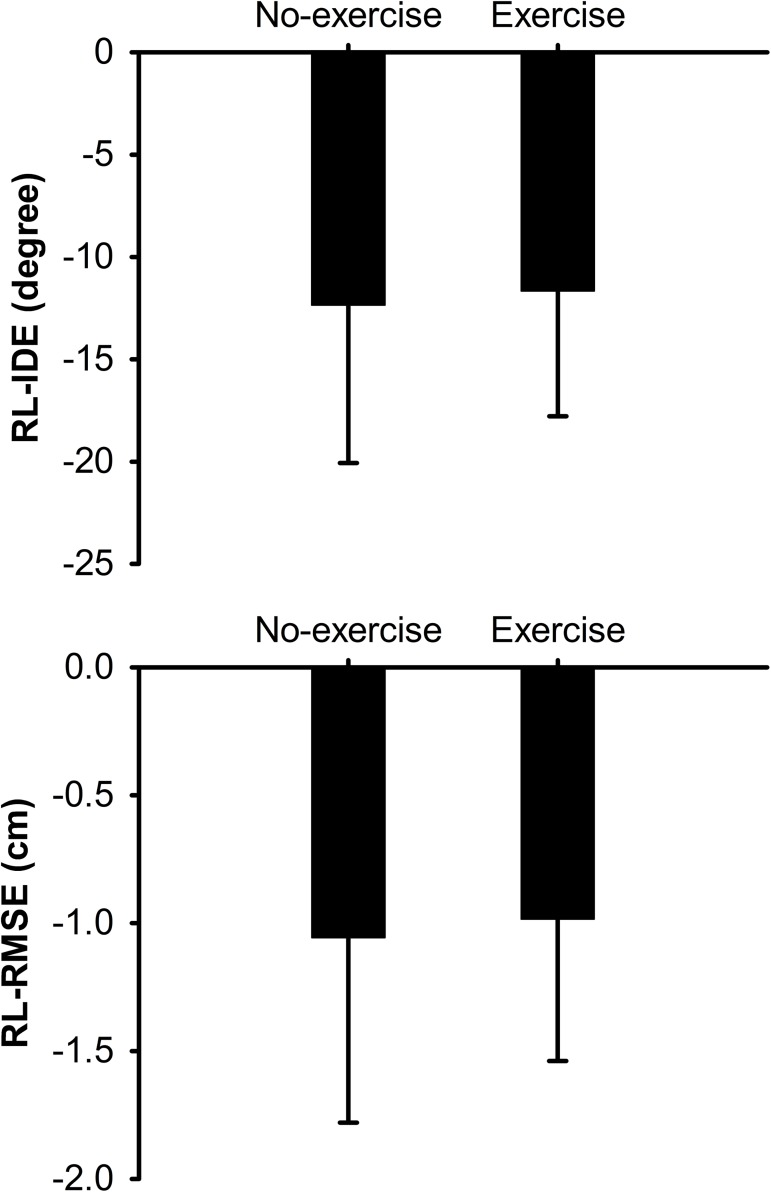
Comparison of the RL for the rVMA during the adaptation set between participants who did and did not perform exercise before the rVMA. RL was calculated for the error variables IDE and RMSE and expressed by mean and SD. *Abbreviations*: IDE = initial directional error; RL = Rate of learning; RMSE = root mean squared error; rVMA = rotational visuomotor adaptation task.

**Fig 5:**
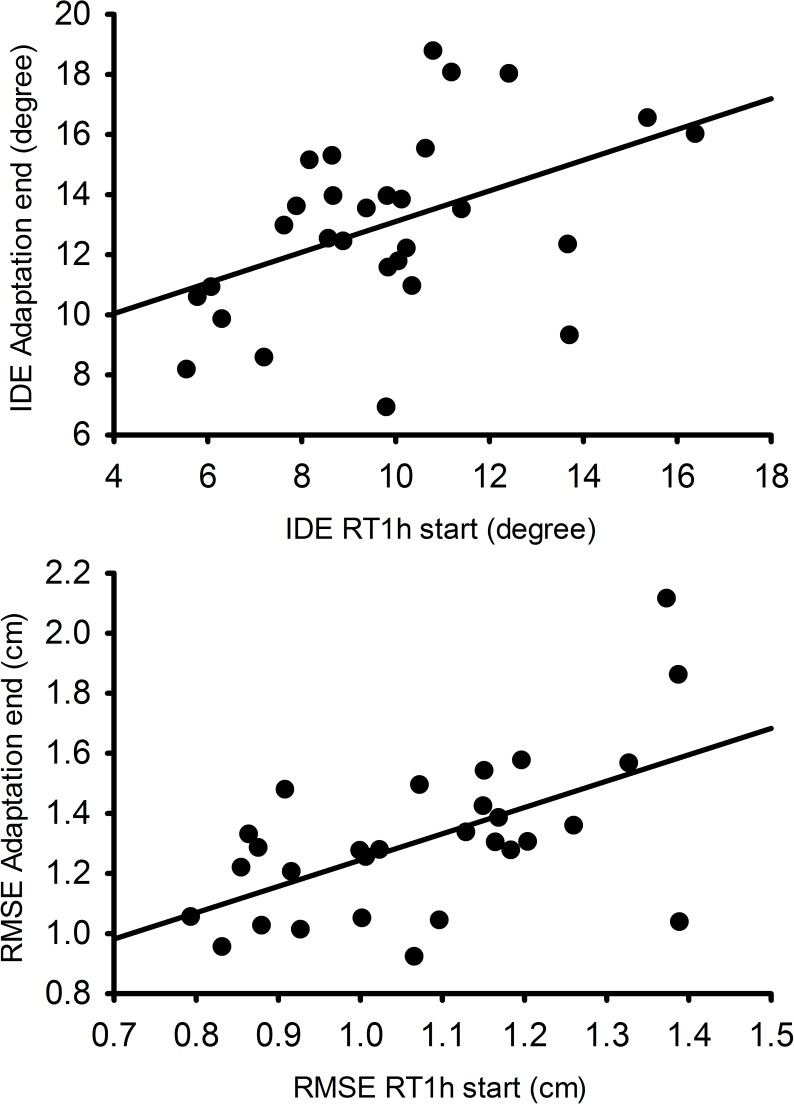
Correlation between error performance at the end of the adaptation and the start of the RT1h of the rVMA. IDE and RMSE mean errors were calculated at the end of the adaptation set (last 32 trials, 4 epochs) and at the start of the RT1h (first 32 trials, 4 epochs). Performance at the end of the adaptation and the start of RT1h were significantly correlated for both error variables: IDE and RMSE. *Abbreviations*: RT1h = retention set at 1h from adaptation set; IDE = initial directional error; RMSE = root mean squared error; rVMA = rotational visuomotor adaptation task.

In the adaptation set we evaluated the impact of iE on the averaged descriptive and error variables, and on the initial error reduction on RL (Table 2). The *t*-tests showed similar results between those who exercised before the rVMA (exercise cohort) and those who did not (no-exercise cohort) for MT (t_(27)_ = -0.579; *p* = 0.568; *d* = 0.199), TD (t_(27)_ = -0.695; *p* = 0.493; *d* = 0.283), and RT (t_(27)_ = -0.631; *p* = 0.533; *d* = 0.229). Means and SDs for the no-exercise cohort were: 163.16 ± 43.49 ms for MT, 7.53 ± 0.39 cm for TD, and 349.57 ± 24.24 ms for RT (see EX–rVMA group values in Table 2). Comparison of the averaged errors also revealed similar performance between cohorts for the IDE (t_(27)_ = -0.385; *p* = 0.703; *d* = 0.154) and the RMSE (t_(27)_ = -0.102; *p* = 0.919; *d* = 0.043) with means in the no-exercise cohort of 16.56° ± 3.45° and 1.56 ± 0.29 cm for IDE and RMSE respectively. Similarly, the Mann–Whitney *U* test showed comparable cohort results regarding the rate of learning for RL-IDE (exercise cohort median = -12.26°; no-exercise cohort median = -10.83°; *U* = 88; *p* = 0.748; *r* = 0.05) and RL-RMSE (exercise cohort median = -0.92 cm, no-exercise cohort median = -0.78 cm; *U* = 82; *p* = 0.551; *r* = 0.11) (Fig 4). These data mean that all participants adapted at a similar rate. Furthermore, taking together, all participants showed that performance at the end of the adaptation was significantly and positively correlated with the beginning of the RT1h (IDE *r* = 0.46, *p* = 0.012; RMSE *r* = 0.578, *p* = 0.001) (Fig 5). These results revealed that the exercise had no significant effects on the movement approach, the error values, or the error decrease rate during the adaptation set.

**Fig 6:**
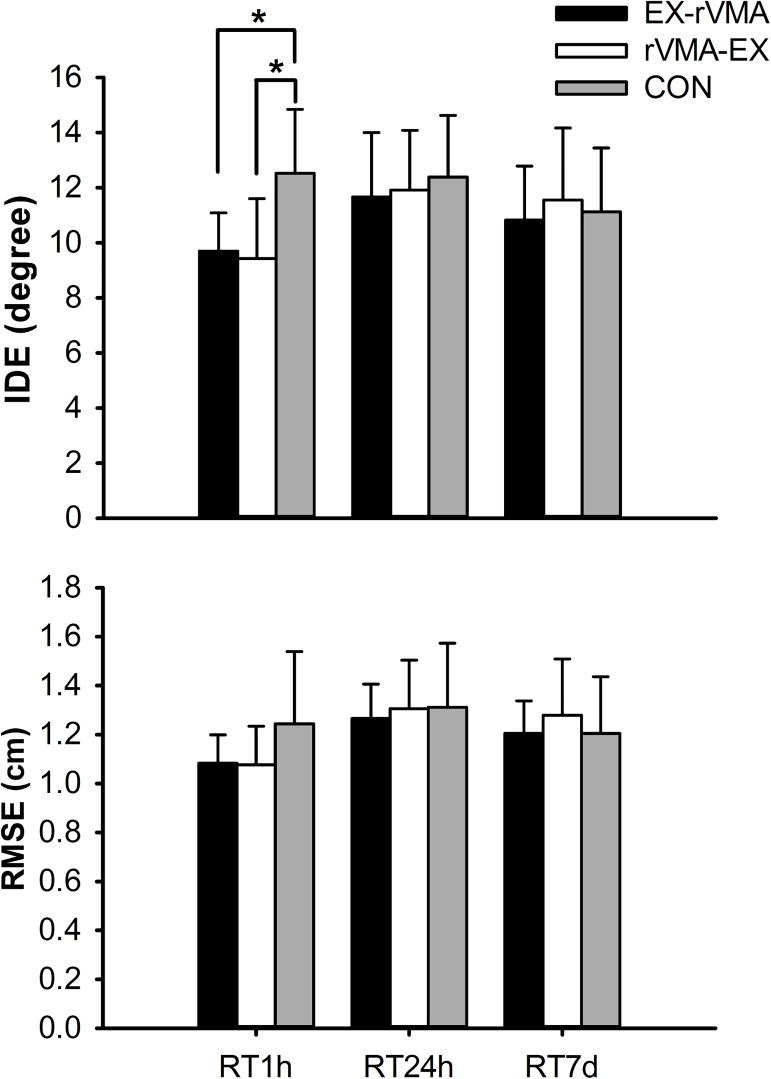
Error values among groups for the rVMA during the retention sets. Error values (mean and SD) are shown for the rVMA during the retention sets (short, RT1h, mid, RT24h, and long-term retention, RT7d). Significant differences between groups are represented by (*). *Abbreviations*: CON = no-exercise group; EX–rVMA = rVMA after exercise group; IDE = initial directional error; RL = Rate of learning; RMSE = root mean squared error; RT = reaction time (shown at 1 h = 24 h = and 7 days); rVMA = rotational visuomotor adaptation task; rVMA–EX = rVMA before exercise group.

Repeated-measures ANOVA was used to assess differences among groups during the retention sets (short-term = 1 h [RT1h]; mid-term = 24 h [RT24h]; and long-term = 7 d [RT7d]) of the rVMA task (Table 2 and Fig 6). There were no significant differences in the interaction between groups and sets regarding MT (F_(4, 52)_ = 2.107; *p* = 0.093; η^2^*p* = 0.139), TD (F_(4, 52)_ = 1.161; *p* = 0.338; η^2^*p* = 0.082), and RT (F_(4, 52)_ = 0.192; *p* = 0.942; η^2^*p* = 0.015). By contrast, significant group × set interactions were found, with a large effect size, for both IDE (F_(4, 52)_ = 30.946; *p* = 0.007; η^2^*p* = 0.233) and RMSE (F_(4, 52)_ = 3.685; *p* = 0.010; η^2^*p* = 0.221). Post hoc analysis only depicted a significant difference for the IDE at RT1h, with both exercise groups (EX–rVMA: *p* = 0.014; rVMA–EX: *p* = 0.007) showing lower error values than the CON group, indicating a positive effect of exercise on the RT1h. No significant differences were found among the groups at RT24h and RT7d.

## Discussion

In this experiment, we sought to assess the effect of a single bout of iE on the adaptation to, and retention of, an rVMA task. We also investigated whether the order of task and exercise presentation produced different retention results. Regarding the adaptation set, there were no differences in the rVMA between those who did and did not exercise before the task, as evaluated by output movement error variables (RL-IDE and RL-RMSE), indicating that exercise did not contribute to improving the RL. Likewise, the overall movement error performance (IDE and RMSE) and descriptive (MT, TD and RT) parameters were not enhanced by the exercise bout. Thus, these results did not support our hypothesis that exercise would have a positive effect on motor adaptation when presented before motor tasks[20,22].

Timing between exercise and task presentation, the task characteristics (type of task and complexity), and the exercise characteristics (type of exercise, duration, and intensity) are some of the factors that have been seen to modulate this exercise-brain function relation [11,31–33].It is possible that the exercise intensity used in the present study may havehindered the possible beneficial effects of exercise for adaptation to the motor task after exercise. Similar results have been obtained by Roig et al. [21], who showed that a bout of high-intensity exercise before practicing a manual tracking task had no impact on adaptation. They proposed that exercise could induce fatigue, thereby hampering the possible benefits of exercise during adaptation by decreasing the accuracy. Considering the similarities between the exercise used in this and in Roig’s study [21], we speculate that fatigue, in addition to excessive arousal, may have caused the observed results in the adaptation set of the rVMA task. However, Mang et al. [22] found that, despite using a similar high-intensity exercise program to the one used by Roig et al. [21], participants who exercised before the motor task adaptation had enhanced performance. Despite this enhanced adaptation, exercise-induced benefits were only observed in the temporal components of the motor task. In the present study, exercise did not boost motor adaptation on any of the spatial or temporal task parameters, possibly because of the moderating effects of task characteristics over exercise-induced benefits [11]. Thus, further research is necessary on the effects of high-intensity exercise on complex motor learning paradigms in task adaptation.

Regarding retention, IDE values were improved during short-term retention (RT1h) in both exercise groups. IDE is thought to reflect the planning of the movement direction, and thus the state of the internal model of the skill [27]. Consequently, we suggest that the performed bout of iE positively affected short-term consolidation and retrieval of the newly formed internal model of the motor skill. Rotational visuomotor adaptation tasks, are known to be dependent on cerebellar function [34]. It is known that acute intense exercise can impact the excitability of cerebellar circuits and that these cerebellar circuits may contribute to the exercise-induced increase in LTP-like plasticity in brain regions like premotor cortex [35]. However, more research is needed to improve our understanding of the mechanisms underlying the observed exercise-related boost on memory.

Although group differences were not confirmed, movement error expressed as the RMSE produced similar results to the IDE. It must be noted that RMSE includes initial movement planning and feedback-guided corrections during the path to the target. The fact that no differences were found among groups for the MT and TD at RT1h may indicate that quick and efficient correction of the trajectory occurred through the use of feedback in the control group. Despite participants in the exercise groups performing better at initial movement planning, feedback-guided corrections in the control group allowed them to correct the initial trajectory deviations to achieve comparable RMSE values to those of the exercise groups.

In contrast to the strengthening of the short-term retention (RT1h), exercise failed to maintain the observed benefits in the retention sets at 24 h (RT24h) and 7 days (RT7d) after adaptation. The IDE and RMSE values were similar for the three groups, indicating that exercise did not affect motor planning or feedback utilization at 24 h or 7 days after adaptation to the skill. Based on previous research [13,21,22], we hypothesized that exercise would positively impact the RT24h and RT7d results. Roig et al. [21] found that a single bout of high-intensity exercise enhanced motor memory retention at 24 h and 7 days after adaptation. In a study by Mang et al. [22], a similar exercise intervention enhanced mid-term retention of motor memory (24 h). It seems that, compared to previous research, we demonstrated the more acute and transient effect of exercise. Three factors could explain why exercise may not induce persistent effects on memory formation: differences in the exercise bout characteristics (i.e., intensity and/or exercise mode), the fitness level of the participants, and the characteristics of the learning task [33]. We will now examine each of these factors separately.

High-intensity exercise may have the potential to facilitate memory consolidation [21,22,36]. However, as seen in previous research, exercise bouts of insufficient intensity (low to moderate intensity) may not be sufficient to improve motor consolidation [20,37]. When compared with studies that succeeded in finding 24 h and 7 day improvements in long-term memory, the exercise intensity in the present study (estimated 85% VO_2_max) may have been too low; for example, Mang et al. [22] used 90% of power output, which was similar to that utilized by Roig et al. [21]. In addition to intensity, there is evidence that the mode of exercise may influence its benefits. Recent reviews propose that cycling produces a greater effect on cognitive performance [31] and long-term memory [14] than running. Accordingly, the running intervention used in the present study could have produced inferior results when compared to those from similar studies that used cycling [21,22]. This hypothesis is somewhat supported by the results of Statton et al. [20], who successfully enhanced motor adaptation through moderate-intensity aerobic running, but failed to induce more long-term benefits in motor retention. Hence the exercise bout used in this study may have been too intense to improve adaptation, but insufficiently intense to enhance the retention at 24 h and 7 days, with retention benefits limited to 1 h after adaptation.

The fitness level of the participants could also have affected the extent of the exercise-induced improvements. Exercise has been reported to exert greater effects on long-term memory when participants have only average fitness levels [14]. Our sample seemed to have higher fitness levels which could explain differences between our results and those from previous studies. When comparing the fitness level of our exercise group (mean estimated VO_2_max: 56.06, range: 43.3–63.6 ml/kg/min) to that of Mang et al. [22] (mean VO_2_peak: 45.36, range: 30.4–63.4 ml/kg/min), it is plausible to consider that fitness level promoted mid-term retention in the study by Mang et al. [22], but promoted short-term retention in our study. However, when compared with the results presented by Roig et al. [21] (mean VO_2_peak: 53.35, range: 44.1–64.1 ml/kg/min), who achieved enhanced mid- and long-term retention, the fitness level was similar to that in the present study. Therefore, it remains unclear how fitness level could moderate the exercise-induced improvements on motor learning. The fitness level homogeneity of our sample limited the possibility to further explore the potential modulating effect of fitness level on the exercise-learning relationship. More research is necessary to increase our knowledge in terms of the effects of exercise on the mechanisms associated with learning improvement and the role of moderators (e.g., fitness level) in this relationship.

In addition to exercise characteristics and fitness level, task characteristics could also have affected the study results. It is known that the effect of exercise on cognitive function [11,31] and long-term memory formation [14] can be modulated by the paradigm of the cognitive or learning task used. In the present study, improvements in short term retention, 1 h from motor adaptation, were specifically observed for a rVMA task utilizing a 60° clockwise rotation. Previous research using motor procedural tasks have been successful in finding exercise-induced benefits on mid- and long-term memory at 24 h and 7 days after exposure [21,22]. However, to our knowledge, this is the first study describing the effect of an exercise intervention on the learning of a complex procedural motor skill that involved a multi-joint and multi-plane movement paradigm. The complexity of the rVMA task could have decreased the effect of exercise, limiting the anticipated mid- and long-term benefits. Moreover, the previous studies by Mang et al. [22] and Roig et al. [21] relied on tasks mainly focused on accuracy. By contrast, participants in our rVMA task were instructed to “move the cursor over the target as fast and as straight as possible”, which required not only accuracy but also speed of processing and execution. Research has differentiated the effects of exercise in speed and accuracy components of cognitive and simple motor tasks, with speed benefiting most from exercise [32]. Depending on the weight of the speed and accuracy components, and based on the results of previous research [32], we propose that the speed–accuracy relationship could alter the effect of exercise on memory consolidation. Therefore, the speed requirements in the execution of the rVMA task may have affected participant’s long-term memory formation. In addition, despite involving some form of motor adaptation, the use of alternative learning paradigms (e.g. implicit sequence learning) in previous studies [22] could explain different results compared to the present research possibly because of the implication of different neural pathways [38], among other factors.

Finally, contrary to what previous research has defined [21], we observed no differences in memory consolidation based on whether exercise was presented before or after the adaptation set. We presume that the initial consolidation stages may equally benefit from exercise regardless of whether it is presented before or after adaptation. However, the null effect of exercise on mid- and long-term retention hinders further speculation on how the order in which exercise is presented may modulate longer delayed effects. More research is needed to confirm whether the presentation order of the exercise relative to practice can trigger different mechanisms, as proposed by Roig et al. [21].

Despite finding an enhanced rVMA retention at 1 h as a consequence of the exercise intervention, our results may be affected by some limitations. It could be though that exercise effects on motor consolidation could be influenced by relearning because of the high amount of trials performed during the retention sets. However, IDE presented a similar trend of findings during the first trials of the RT1h set, compared to the overall set performance (see graph B in S1 Fig). Furthermore it seems that in comparison to other studies [21,22], the reduced exercise intensity and the mode of exercise (i.e., running) might have compromised the exercise-induced benefits on mid- and long-term retentions. Likewise, it is possible that the high fitness level of participants in the present study could have altered the mid- and long-term effects. Therefore, to confirm the mid- and long-term benefits seen in previous studies, there would be a need to use a higher exercise intensity (90% of VO_2_max) and to include a population with regular fitness levels. Furthermore, the lack of neurochemical assessments limits our ability to comment on the mechanisms that may trigger the consolidation enhancement induced by exercise. In future research, the collection of blood samples to examine changes in neurotransmitters and trophic factors concentrations may help clarify the mechanisms underlying the exercise-induced enhancement of memory consolidation.

## Conclusions

In conclusion, a single bout of iE enhanced consolidation of an rVMA task, as expressed by improved retention at 1 h after task adaptation. Moreover, the order in which the exercise and the learned task were presented yielded similar benefits in retention at 1 h. However, we cannot reject the possibility of a long-term effect of exercise and task presentation order, because the exercise characteristics and fitness levels of the participants may have limited the benefits on mid and long-term retentions. Contrary to our expectation, exercising before a task practice did not improve the learning rate of the motor skill, probably because the exercise intensity was too high and there was a possibility of fatigue. Our results add evidence to the practical uses of exercise in learning and memory, but indicate that further research is needed to improve our understanding of how different exercise protocols affect procedural learning tasks. Moreover, to explore the effect of acute exercise on learning in different populations, future studies should aim to include participants with different fitness levels and ages.

## Supporting information

S1 Fig Exercise effects on motor adaptation and motor retention 1h. **(A)** IDE Performance is presented at the start and at the end of the adaptation (average of first and last 32 trials, respectively). IDE significantly decreased across all three groups as an effect of time, from start to end of the adaptation set. **(B)** IDE at the start (average of first 32 trials) and overall average IDE during the retention set at 1 hour (RT1h) are presented. Similar trends were observed between the RT1h start and the RT1h overall, which could indicate that exercise effects may begin from the start of RT1h. Additionally, performance level in all groups at the RT1h start was only slightly higher compared to their performance during adaptation end, meaning that some consolidation occurred during the 1 h rest period. *Abbreviations*: IDE = initial directional error; RT1h = Retention set at 1 hour; EX–rVMA = rVMA after exercise group; rVMA-EX = rVMA before exercise group; CON = no-exercise group.
(TIFF)
