# Supplementary figures and images for: Enhancing consolidation of a rotational visuomotor adaptation task through acute exercise
Source: PLoS One. 2017 Apr 13;12(4):e0175296. doi: 10.1371/journal.pone.0175296 (PMC5391069; doi:10.1371/journal.pone.0175296)

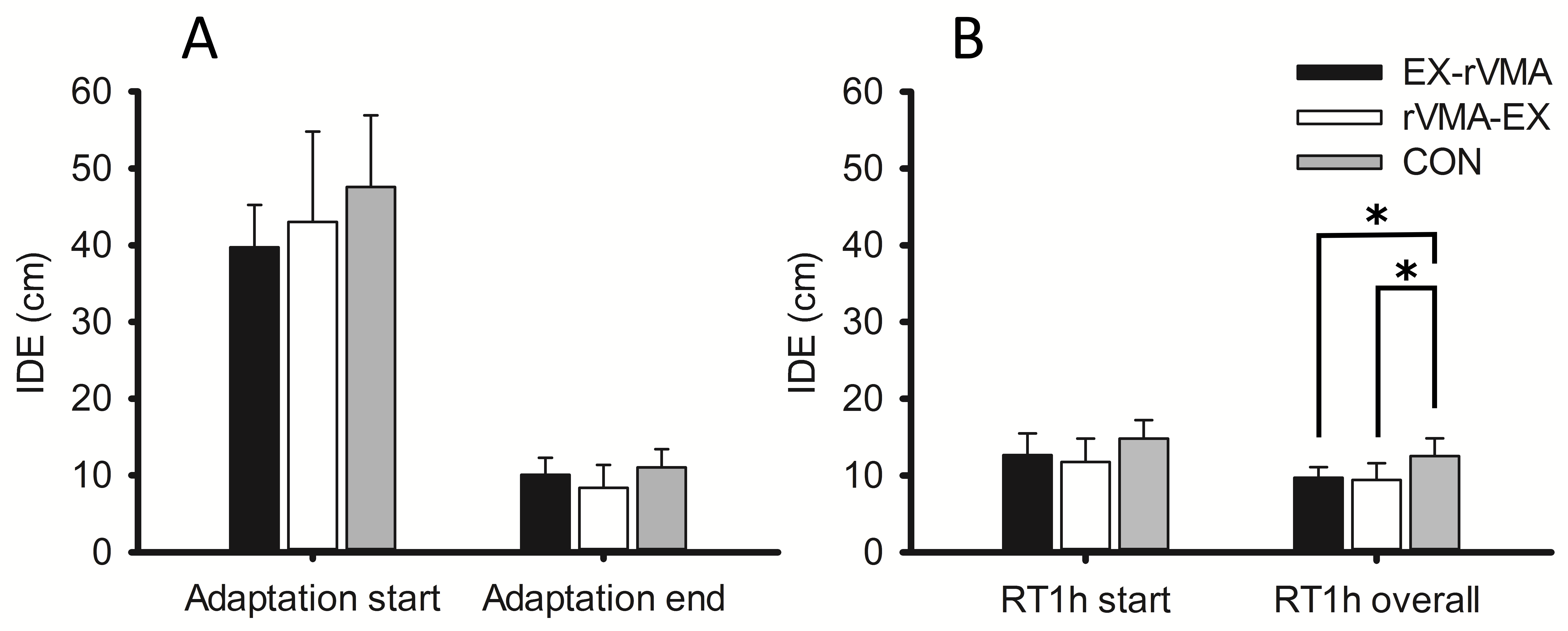

Supplement: S1 Fig — (A) IDE Performance is presented at the start and at the end of the adaptation (average of first and last 32 trials, respectively). IDE significantly decreased across all three groups as an effect of time, from start to end of the adaptation set. (B) IDE at the start (average of first 32 trials) and overall average IDE during the retention set at 1 hour (RT1h) are presented. Similar trends were observed between the RT1h start and the RT1h overall, which could indicate that exercise effects may begin from the start of RT1h. Additionally, performance level in all groups at the RT1h start was only slightly higher compared to their performance during adaptation end, meaning that some consolidation occurred during the 1 h rest period. Abbreviations: IDE = initial directional error; RT1h = Retention set at 1 hour; EX–rVMA = rVMA after exercise group; rVMA-EX = rVMA before exercise group; CON = no-exercise group. (TIFF) [file pone.0175296.s001.tiff]
